# Male X-linked genes in *Drosophila melanogaster* are compensated independently of the Male-Specific Lethal complex

**DOI:** 10.1186/1756-8935-6-35

**Published:** 2013-10-26

**Authors:** Philge Philip, Per Stenberg

**Affiliations:** 1 Molecular Biology, Umeå University, Umeå 901 87, Sweden; 2 Computational Life Science Cluster (CLiC), Umeå University, Umeå 901 87, Sweden

**Keywords:** Buffering, Dosage compensation, Male-Specific Lethal complex

## Abstract

**Background:**

In organisms where the two sexes have unequal numbers of X-chromosomes, the expression of X-linked genes needs to be balanced not only between the two sexes, but also between X and the autosomes. In *Drosophila melanogaster*, the Male-Specific Lethal (MSL) complex is believed to produce a 2-fold increase in expression of genes on the male X, thus restoring this balance.

**Results:**

Here we show that almost all the genes on the male X are effectively compensated. However, many genes are compensated without any significant recruitment of the MSL-complex. These genes are very weakly, if at all, affected by mutations or RNAi against MSL-complex components. In addition, even the genes that are strongly bound by MSL rely on mechanisms other than the MSL-complex for proper compensation. We find that long, non-ubiquitously expressed genes tend to rely less on the MSL-complex for their compensation and genes that in addition are far from High Affinity Sites tend to not bind the complex at all or very weakly.

**Conclusions:**

We conclude that most of the compensation of X-linked genes is produced by an MSL-independent mechanism. Similar to the case of the MSL-mediated compensation we do not yet know the mechanism behind the MSL-independent compensation that appears to act preferentially on long genes. Even if we observe similarities, it remains to be seen if the mechanism is related to the buffering that is observed in autosomal aneuploidies.

## Background

In organisms where females have two X-chromosomes and males have only one, some type of dosage compensation is needed. The expression of X-linked genes needs to be balanced not only between the two sexes, but also between X and the autosomes, in order to maintain the balance of metabolic networks [1-3]. In *Drosophila melanogaster*, this is achieved by an approximately 2-fold increase in expression of the genes on the male X-chromosome. It is commonly stated in the literature that the Male-Specific Lethal (MSL) complex is responsible for this 2-fold increase by binding to expressed genes on the male X [4-8]. The MSL-complex consists of five protein components, namley the H4K16 histone acetyltransferase Male absent on the first (MOF), Maleless, Male-Specific Lethal 1 (MSL1), Male-Specific Lethal 2 (MSL2), and Male-Specific Lethal 3 (MSL3), and two non-coding RNAs (*roX1* and *roX2*) [9,10]. The components of the MSL-complex clearly have important functions since mutations are male-specific lethal. However, many (10–25%) of the expressed genes on the male X are not bound by the complex [11-14]. There have been different explanations for this; the most common explanation has been that these unbound genes are only transiently bound by the complex and thus binding escapes detection [15]. Although it has generally been assumed that the genes that are unbound but still expressed are dosage compensated, this assumption has not been investigated thoroughly on a genome-wide level. Zhang et al. found that genes on the X-chromosome were, on average, dosage compensated approximately 1.35-fold by an MSL-dependent mechanism, irrespective of the gene dose, in a male cell-line [16]. It has been speculated that the remaining compensation, up to two-fold, could be mediated by a more general buffering mechanism acting on all genomic regions that are present in single copy [16-19]. The idea that the MSL-complex is not responsible for the bulk of chromosome X dosage compensation has been previously proposed by Birchler et al. [20-22], suggesting that an “inverse dosage effect” causes an up-regulation of the single male X-chromosome mainly through the loss of negative regulators. However, to date, no study has attempted a genome-wide scale correlation between compensation and the binding of the MSL-complex at the individual gene level.

If the MSL-complex is the mediator of the full, 2-fold, dosage compensation, we would expect to observe an approximately 2-fold reduction in the expression of genes on the X-chromosome if components of the complex are removed. Arguing against this prediction, several studies have shown that expression of male X-linked genes is only reduced to about 80% of the normal value after RNAi knock-down or mutation of MSL-complex components [5,6,23,24].

In this paper, we use publicly available data which we combine and present in novel ways in order to investigate the proportion of expressed genes on the male X that are dosage compensated. We also analyze how much the dosage compensated genes are dependent on the MSL-complex.

## Results and discussion

### Most expressed genes on the male X are dosage compensated

**Figure 1:**
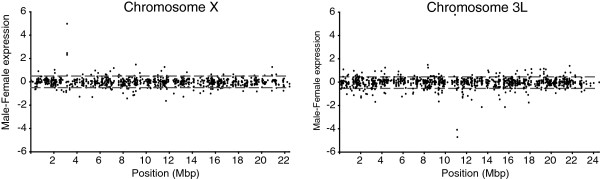
**Male minus female expression in salivary glands (log**_**2**_**) of all genes expressed in both sexes along chromosomes X and 3L.** Dotted grey lines indicate ± one standard deviation from the mean of chromosome 3L.

We first set out to investigate how many of the expressed genes on the male X-chromosome are dosage compensated. To do this, we used the (to our knowledge) only available set of genome wide expression data from a single tissue (salivary glands) where both males and females were studied separately [8]. It is important to perform this analysis on a sample including as few different cell types as possible since we would otherwise be unable to distinguish gene expression differences between the sexes from, for example, variation in cell type proportions. Similarly, we cannot use cell-lines as male and female cell-lines could have very different origins. Since unexpressed genes or genes expressed below the detection limit of the technique (in this case array) will appear to be dosage compensated, we excluded all genes not expressed in males and/or females, using the same approach as in previous studies [18,25,26]. We next plotted the male/female expression ratio across the major chromosome arms (chromosome X and 3L are shown in Figure 1, the other major chromosome arms are shown in Additional file 1: Figure S1) and calculated the ratio mean and standard deviation (the male/female ratio appears to be normally distributed on all chromosomes). Interestingly the chromosomes show a similar mean and standard deviation (mean -0.044 to -0.003, SD 0.33 to 0.51). The X-chromosome seems to be well compensated (mean -0.020) and shows low variation in the expression ratio (SD 0.38).

We then defined dosage compensated genes as genes on chromosome X that are expressed in both males and females and where the log_2_ ratio was within one standard deviation of the mean for an autosomal chromosome; for this study we used chromosome 3L (> - 0.55, <0.47). According to this definition, 90.4% of all expressed genes (n = 882) on the X-chromosome are dosage compensated (Figure 1), a value which is comparable to those of the autosomes where 88.2% to 91.1% of the genes are expressed at the same levels in males and females.

Based on these results, we conclude that most genes on the male X-chromosome are effectively compensated and that there are similar numbers of differentially expressed genes when comparing males and females across all major chromosome arms. Although this effect has not been formally demonstrated previously, it is in line with current opinion in the field. Expression levels in males and females have been compared in other studies [24,27]. However, these comparisons were done using whole flies, making it impossible to separate dosage compensatory effects acting on individual genes from differences in expression between tissues. Moreover, such studies (or studies of whole embryos) do not allow direct comparison of expression effects at an individual gene level with binding levels of the MSL-complex.

### About 15% of expressed genes on male X are dosage compensated but unbound by MSL and have significantly reduced levels of H4K16 acetylation

**Figure 2:**
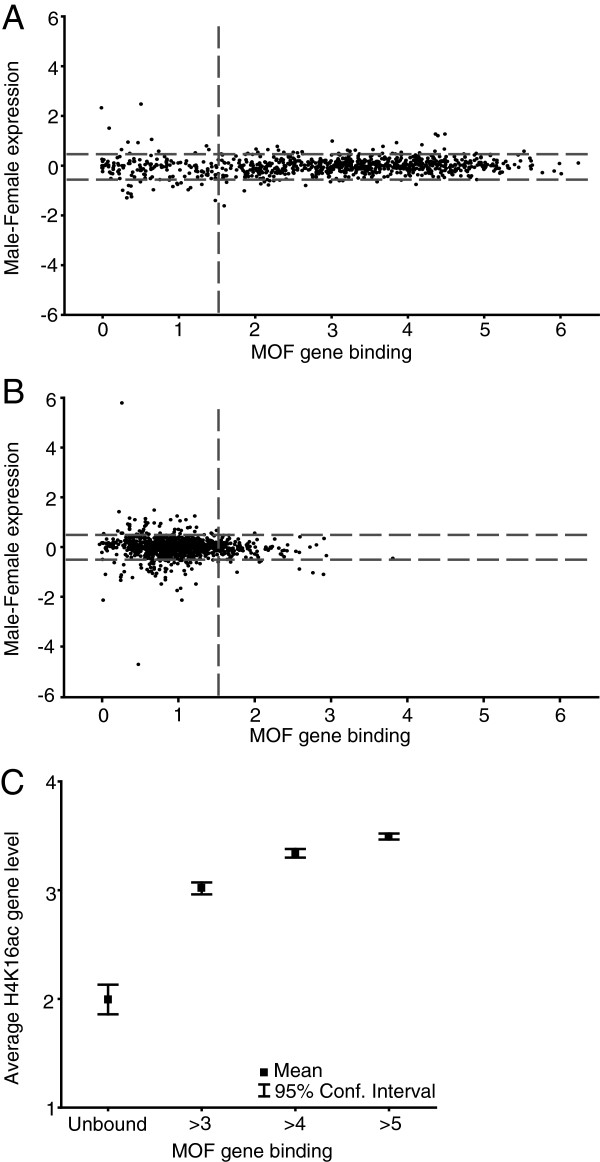
**A significant part of the genes on the X chromosome are dosage compensated, unbound by MOF and not H4K16 acetylated in salivary glands.** Male minus female expression (log_2_) of all genes expressed in both sexes plotted versus MOF gene binding values on **(A)** the X-chromosome and **(B)** chromosome 3L. Horizontal dotted grey lines indicate ± one standard deviation from the mean expression ratio for chromosome 3L and vertical lines the mean MOF binding on chromosome 3L plus one standard deviation. **(C)** Average H4K16ac in four non-overlapping groups with different levels of MOF binding, (Unbound n = 122, >3 n = 220, >4 n = 227, >5 n = 227).

Various figures have been reported for the number of genes that are expressed but not bound by MSL [11-14]. To determine how many of the compensated genes are significantly bound by the MSL-complex we used available MOF binding data from male salivary glands [28]. MOF binds to both the promoters and the gene bodies of MSL-targeted genes on the X-chromosome [26,29,30]. After determining the extent of binding to all gene bodies excluding the first 200 bp of exon sequence (see Methods), we plotted the male/female expression ratio versus MOF binding for all chromosomes (chromosome X and 3L are shown in Figure 2A,B).

To determine which genes undergo binding by MOF to the gene body, we calculated the mean and standard deviation of MOF binding to expressed genes on chromosome 3L (MOF binding values were found to be normally distributed on 3L). Genes on X with a binding value higher than the 3L mean plus one standard deviation (mean = 1.00, SD = 0.48) were considered to be bound. This cut-off probably identifies some unbound or very weakly bound genes as bound, since 147 genes on chromosome 3L would be considered bound by this definition. It is known that MOF is also bound to autosomes as part of the Non-Specific Lethal complex [31], but since this binding is to the promoters of genes it will not influence our calculated gene binding values for MOF (see Methods). Using this definition, we find that 122 genes on the male X-chromosome are dosage compensated although not bound at any detectable level by the MSL-complex. The fact that many expressed genes are not bound by the MSL-complex has been reported several times. However, the level to which these genes are compensated has been unknown [29,32,33]. The usual explanation for the lack of MSL binding to these expressed genes has been that they only transiently interact with the complex and they do show H4K16 acetylation marks. However, when we look at the H4K16ac data from male salivary glands [8] in the gene bodies of all dosage compensated X-linked genes, we see drastically reduced levels of acetylation of the genes that we classified as unbound but still compensated (Figure 2C). Although we cannot exclude the possibility of the MSL-complex influencing these genes on the basis of these results, we argue that it is very unlikely.

The data we are using is from salivary glands which have multiple copies of each chromosome. It is known that parts of the genome are under-replicated in salivary glands and the female X-chromosome have more such regions when compared to the male X-chromosome [34,35]. It is therefore possible that MSL unbound genes in our data set will appear to be compensated, when in fact they may have similar copy numbers in males and females. To investigate this, we first counted how many expressed and compensated genes map to the under-replicated regions on the X-chromosome reported by Makunin et al. [36]; 27% of the MSL unbound, expressed and compensated genes map to under-replicated regions, which is very similar to the 28% of strongly bound (MOF binding value >5), expressed and compensated genes that map to under-replicated regions. In addition we calculated the copy number ratio between males and females of all compensated genes using the input reads of the MOF ChIP-seq experiments. The male and female input samples varied in sequencing depth but we observed that on average the X-linked genes clearly have a lower male to female ratio when compared to the autosomes (Man-Whitney U Test, *P* <0.05). The unbound compensated genes did not have more similar copy number in males versus females. In this analysis we saw a weak trend that the more MOF bound the higher the male/female copy number ratio. We therefore conclude that the compensation of the MSL unbound genes is not caused by similar copy numbers in males and females.

### Genes unbound by MSL but still compensated are not affected by MSL RNAi

In order to determine whether the genes identified as unbound (or bound below the detection limit) by MSL are in fact independent of the MSL-complex, we turned to MSL-complex component RNAi knock-down and mutant data available from S2 cells and male larvae, respectively. Although these data sets are not from salivary glands, we and others have shown that MSL-complex binding is extremely similar between different cell types [13,26,32] (Additional file 2: Figure S2). Although the MSL binding levels of some genes are likely to differ between, for example, S2 cells and salivary glands, we believe that comparison of data from these sources will be meaningful (we exclude genes that are not expressed in the wild-type or mock RNAi).

**Figure 3:**
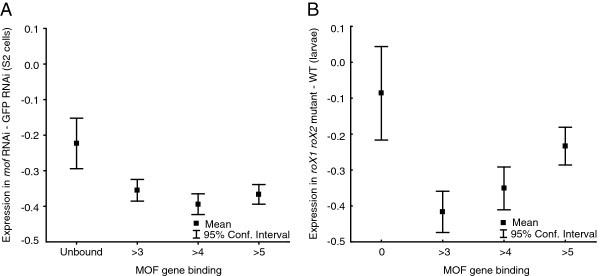
**MOF unbound genes in salivary glands are less affected in MSL-complex mutants/knock-downs. (A)** Expression in S2 MOF RNAi lines (log_2_ expression of MOF RNAi-GFP RNAi, Unbound n = 86, >3 n = 208, >4 n = 222, >5 n = 226). **(B)** Expression in *roX1 roX2* mutant larvae (log_2_ expression of *roX1 roX2*-WT, Unbound n = 100, >3 n = 212, >4 n = 205, >5 n = 205).

When we correlate the effect of RNAi knock-down or mutations in MSL-complex components with MOF binding to compensated genes, we see that the expression of genes that are unbound but still compensated is very weakly affected by MSL (Figure 3; Additional file 3: Figure S3). In the mutant data sets from male larvae, the unbound genes are virtually unaffected, and in the RNAi experiments these genes show a weak reduction in expression (significantly different from autosomes, four RNAi data sets, Man-Whitney U Test, all *P* <0.05). The small effect on the unbound genes in the RNAi experiments could indicate that they are weakly bound (below what we can detect with ChIP-seq) or that these effects are secondary gene network effects. We note that the most strongly bound genes show less reduction in expression in some experiments (Additional file 3: Figure S3). In the RNAi knock-down experiments this could be due to the fact that knock-down is not complete and that weakly bound genes will lose the MSL-complex before those that are more strongly bound. If so, that would make it unlikely that the genes we defined as unbound have weak interactions with the MSL-complex as those should be lost first. Unbound genes also score lower than bound genes (Man-Whitney U Test, all *P* <0.05) on sequence signatures previously found to be enriched in MSL bound genes in S2 cells [26], further supporting that unbound expressed genes do not recruit MSL. We note that the genes which show the strongest reduction in expression are still expressed at about 80% of wild-type levels. We conclude that a significant proportion of dosage compensated genes on the male X-chromosome is compensated by a mechanism independent of MSL. A few such genes have been identified in earlier studies [21,22,37-41], but in salivary glands, these genes (such as *runt, giant* and *buttonhead*) are either not expressed according to our definitions, or, if expressed, bound by MOF.

### MSL independent mechanisms probably play an important role in compensating the male X-chromosome

**Figure 4:**
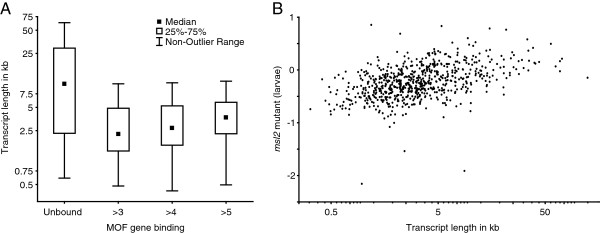
**Long genes are less dependent on the MSL-complex. (A)** Average transcript length in four non-overlapping groups of expressed genes with different levels of MOF binding in salivary glands (Unbound n = 122, >3 n = 220, >4 n = 227, >5 n = 227). **(B)** Expression in *msl2* mutant male larvae (relative to wild-type) plotted versus transcript length.

An MSL-independent mechanism probably also plays a significant role for MSL-bound genes, as the latter are only mildly affected by the loss of MSL-complex components. This was previously observed by (for example) Zhang et al. [16], and could be explained either by the presence of a general buffering mechanism, which recognizes genomic regions present in fewer copies and which acts on all chromosomes [18,25], or by gene network effects [42]. The general buffering of autosomal deficiencies reported by Lundberg et al. showed stronger buffering of long genes [18]; if the same mechanism also acts on the X-chromosome, we expect that long genes would be less MSL dependent. We found that unbound genes are on average much longer than bound genes (Figure 4A) suggesting that long genes are less dependent on the MSL-complex.

If this mechanism applies, we would expect long bound genes to be less affected by reduction or loss of the MSL-complex. When long genes (>10 kb, the approximate median length of unbound genes) are compared to short genes (<10 kb), MSL-bound long genes are less affected by RNAi/mutation of MSL components (five data sets, Man-Whitney U Test, all *P* <0.05), with the exception of the MSL-3 RNAi which was investigated by Kind et al. [29]. The differences between long and short genes are most prominent in the *msl2* and *roX1 roX2* mutant datasets. For bound genes, there is a correlation between transcript length and expression in *msl2* mutants (R = 0.48, *P* <0.05, Figure 4B). We conclude that long X-linked genes are less, if at all, affected by loss of the MSL-complex.

The finding that long genes are less dependent on MSL for dosage compensation is intriguing and is in line with the fact that it has previously been shown that MSL-bound genes are mainly housekeeping genes [12]. We therefore looked at the genes that are unbound but still compensated and found that only 29% of them are classified as housekeeping (according to the definition in [25]) compared to 70% of the bound genes. Kharchenko et al. found that long genes are preferentially found in regions enriched for the enhancer type of chromatin (chromatin state 3) [43]. We speculate that long genes are more dynamically regulated and are less dependent on the MSL-complex for compensation of their expression in males. The binding of MSL to some long genes is probably due to their proximity to high affinity sites. Unbound long genes (>10 kb) are on average positioned 48 kb from a high affinity site (defined by combining the sites reported by Alekseyenko et al. [7] and Straub et al. [44]) while bound long genes are on average 19 kb away.

Recently, two contradictory studies tried to identify the mechanism behind MSL-dependent compensation [8,45]. The results of Larschan et al. indicated enhanced elongation efficiency, but it was later questioned whether enhanced elongation efficiency on the X-chromosome could be demonstrated using the same data [45]. When chromosome X is compared to individual autosomes, no enhanced elongation efficiency can be seen [8,46]. Conrad et al. provided evidence for increased RNA Pol2 initiation on MSL-bound genes [8]. Recently, problems with the analysis pipeline applied to these data have been pointed out [47-49]. However, even after correcting for the pre-processing issues, an approximately 20% increase of Pol2 initiation on MSL-bound genes was observed [47-49]. This is compatible with an MSL-dependent mechanism that recruits Pol2 and with the fact that the loss of MSL results in only an approximately 20% drop in expression. Although additional evidence is needed, it has been proposed that the MSL-complex sequesters MOF from autosomes [21,22] and constrains its activity on the X-chromosome [20,50]. Clearly, the MSL-complex has an essential function, as mutations affecting its components are male-specific lethal, and we speculate that this function is to enhance the expression of genes that lack effective feed-back regulation; these seem to be mostly short housekeeping genes. Although the mechanisms of the MSL independent compensation mechanism remain elusive, they clearly make a significant contribution to dosage compensation of the X-chromosome and merit more focused investigation.

## Conclusions

The Drosophila male X-chromosome is extremely well compensated and a significant number of genes are compensated without being bound by any detectable levels of MSL-complex. Taken together with the fact that even the most strongly affected genes (those with robust MSL binding) in MSL mutants/knock-downs are still expressed at about 80% of wild-type (50% is expected if the MSL-complex is the only player in dosage compensation), the majority of the dosage compensation of all male X-linked genes is likely mediated by an MSL-independent mechanism. Similar to the MSL-mediated part of dosage compensation, we do not yet know the mechanism behind the MSL-independent compensation other than that it appears to act preferentially on long genes. We hope that future studies not exclusively focused on the MSL-complex will shed light on these important MSL-independent mechanisms.

## Methods

### Micro-array expression data analysis

Gene expression levels in wild-type, RNAi, and mutant samples were computed from Affymetrix .CEL files of raw gene expression data using Robust Multi-Array Average [51] with the Bioconductor “affy” package [52]. Each probeset was then mapped to the genomic Release 5 coordinates using the latest library files from Affymetrix. Only probeset mapping to a single gene were included in this study (in the main text these are referred to as genes) and the medians of biological replicates were used for downstream analysis. We defined expressed genes as those with expression values of 6 or more (log_2_) as previously described [18,25,26]. The following datasets were used for the analysis: (male, female wild-type – E-MEXP-3506, *mof, msl1, msl3* RNAi – E-MEXP-1505, *msl2* RNAi – http://compbio.med.harvard.edu/Supplements/GD05.html, *msl2* mutant – GSE12054, *roX1 roX2* mutant – GSE3990).

### ChIP data analysis

Reads from MOF and H4K16ac immunoprecipitation (IP) and input samples (E-MTAB-911) were aligned against the *Drosophila melanogaster* reference sequence (release 5) using the Bowtie software package [53], mapping reads only to unique locations in the genome (parameters: -n 2 –k 1 --best). Read counts per nucleotide were calculated and median smoothed using a window size of 10 bp, and smoothed read counts were extracted at intervals of 10 bp across the genome. For each position in the IP sample with at least one mapped read, a ratio of IP-input (on a log_2_ scale) was calculated. If the read count in the input was below the read-count mean (in the input sample) it was set to the mean. If the input mean was below four the minimum input value was set to four (to avoid division by near-zero values). All ratio values were then adjusted by reducing each value by the median of the ratios. This linear adjustment was carried out in order to compensate for differences in IP and input sequencing depth. Ratio values were further median smoothed using a window size of 200 bp. Windows with fewer than 10 data points were discarded. Gene binding values for MOF and H4K16ac from salivary glands were calculated by first extracting all data points (at 10 bp resolution) within annotated exons of each gene. If the number of data points for a given gene exceeded 10, they were sorted and the gene binding value calculated as the mean of the 50% highest values as described by Johansson et al. [46], with the exception that here we excluded the first 200 bp exonic sequence of each gene. Introns were excluded since both MOF and H4K16ac are enriched in exons. Using the top 50% of the exon values makes the mean less sensitive to alternatively spliced exons. We exclude genes with fewer than 10 data points to avoid uncertain binding values from short genes. We also calculated gene binding values for MOF, MSL1, and MSL3 in S2 cells from data (E-MEXP-1508) in the study by Kind et al. [29].

To calculate copy number ratio for each gene between males and females, read counts per nucleotide in male and female input samples were first median smoothed using a window size of 1 kb and smoothed read counts were extracted at intervals of 10 bp across the genome. All such 10-step positions within compensated genes were averaged and a male/female ratio (on a log_2_ scale) was calculated.

### Statistical analysis and visualization of data

All statistical analyses and visualizations were performed using Statistica (Statsoft, USA) and Evince (UmBio, Sweden). Transcript length was calculated, using Flybase annotation Release 5.43 [54], as the difference between Gene Start and End positions. To calculate the distances between genes and High Affinity Sites (HAS), we used 188 HAS defined by the union of the HAS reported by Alekseyenko et al. [7] and Straub et al. [44]. Before normalization, all data sets were tested for skewness as described by Landfors et al. [55].

## Abbreviations

HAS: High affinity sites; IP: Immunoprecipitation; MOF: Male absent on the first; MSL: Male-specific Lethal; MSL1: Male-specific Lethal 1; MSL2: Male-specific Lethal 2; MSL3: Male-specific Lethal 3.

## Competing interests

The authors declare they have no competing interests.

## Authors’ contributions

PP and PS designed the study, performed the analysis and wrote the paper. Both authors read and approved the final manuscript.

## Supplementary Material

Additional file 1: Figure S1 Male minus female expression (log_2_) of all dosage compensated genes plotted versus MOF gene binding values on the X chromosome (salivary gland data). Coloring from red (strong) to dark blue (weak) based on MOF, MSL-1 and MSL-3 gene binding values in the S2 cell-line. All data are in log_2_. Click here for file

Additional file 2: Figure S2 Expression of genes with variable MOF gene binding values in A) *msl2* mutant versus wild type larvae (Unbound n = 93, >3 n = 208, >4 n = 211, >5 n = 202), B) *msl2* RNAi versus control RNAi in S2 (Unbound n = 69, >3 n = 205, >4 n = 222, >5 n = 224), C) *msl1* RNAi versus control RNAi in S2 (Unbound n = 86, >3 n = 208, >4 n = 218, >5 n = 223), and D) *msl3* RNAi versus control RNAi in S2 (Unbound n = 86, >3 n = 208, >4 n = 222, >5 n = 226). All data are in log_2_. Click here for file

Additional file 3: Figure S3 Male minus female expression in salivary glands (log_2_) of all genes expressed in both sexes along chromosomes 2L, 2R and 3R. Click here for file
