# Supplementary figures and images for: Male X-linked genes in Drosophila melanogaster are compensated independently of the Male-Specific Lethal complex
Source: Epigenetics Chromatin. 2013 Oct 26;6:35. doi: 10.1186/1756-8935-6-35 (PMC4176495; doi:10.1186/1756-8935-6-35)

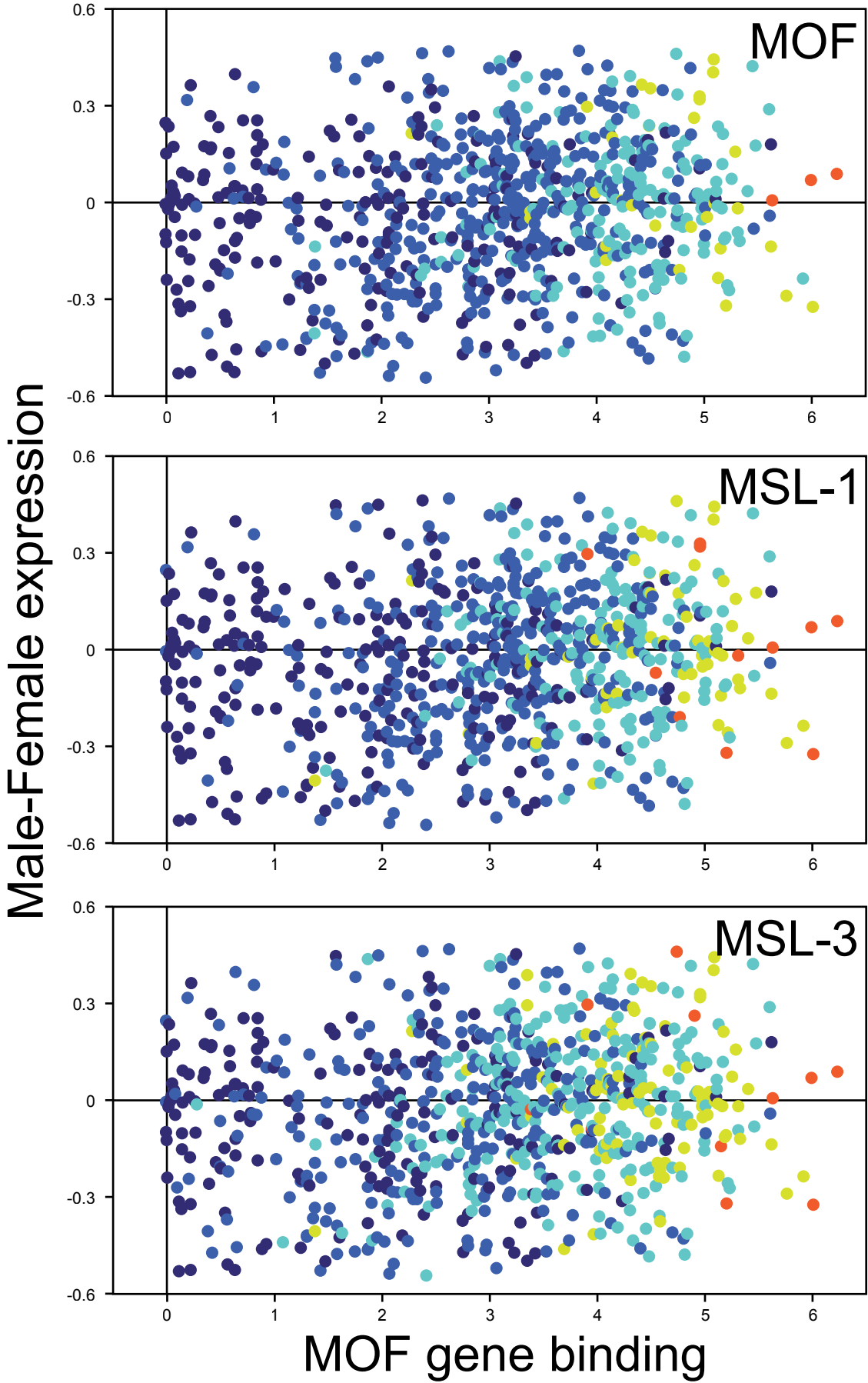

Supplement: Additional file 1: Figure S1 — Male minus female expression (log2) of all dosage compensated genes plotted versus MOF gene binding values on the X chromosome (salivary gland data). Coloring from red (strong) to dark blue (weak) based on MOF, MSL-1 and MSL-3 gene binding values in the S2 cell-line. All data are in log2. [file 1756-8935-6-35-S1.pdf]

A

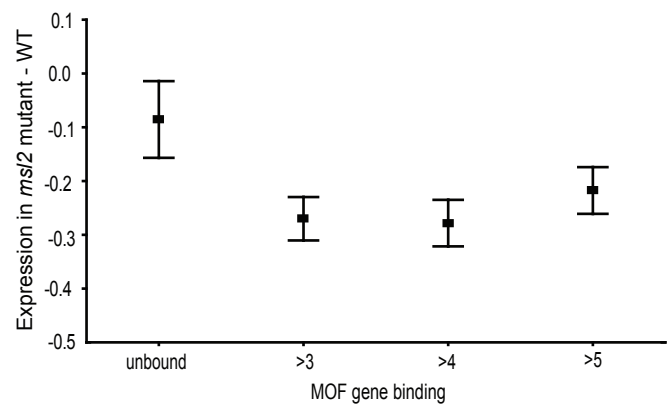

B

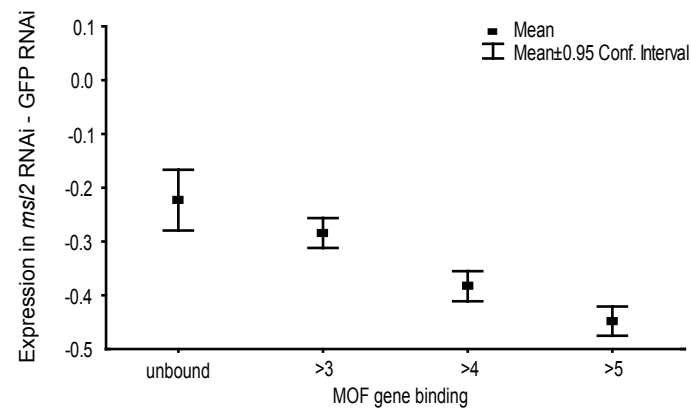

C

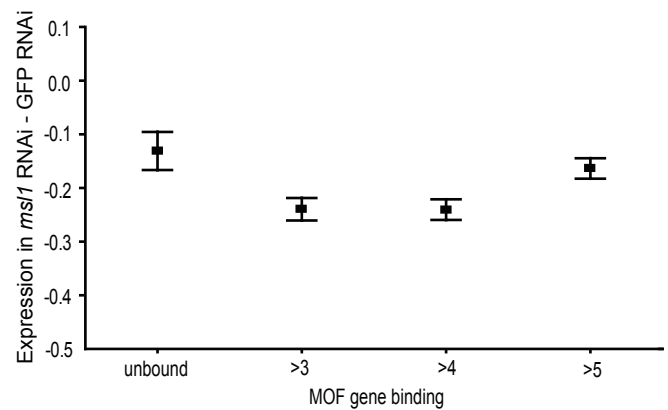

D

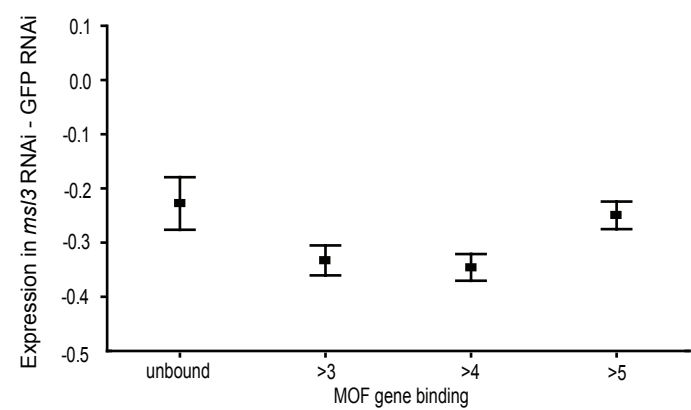

Supplement: Additional file 2: Figure S2 — Expression of genes with variable MOF gene binding values in A) msl2 mutant versus wild type larvae (Unbound n = 93, >3 n = 208, >4 n = 211, >5 n = 202), B) msl2 RNAi versus control RNAi in S2 (Unbound n = 69, >3 n = 205, >4 n = 222, >5 n = 224), C) msl1 RNAi versus control RNAi in S2 (Unbound n = 86, >3 n = 208, >4 n = 218, >5 n = 223), and D) msl3 RNAi versus control RNAi in S2 (Unbound n = 86, >3 n = 208, >4 n = 222, >5 n = 226). All data are in log2. [file 1756-8935-6-35-S2.pdf]

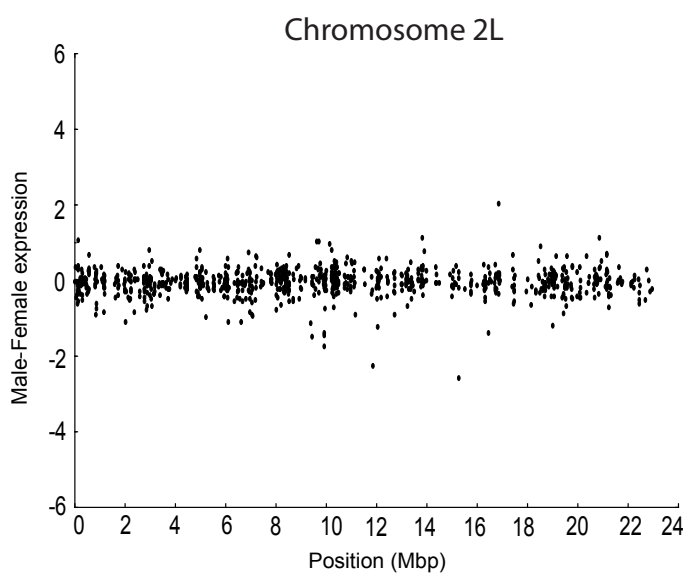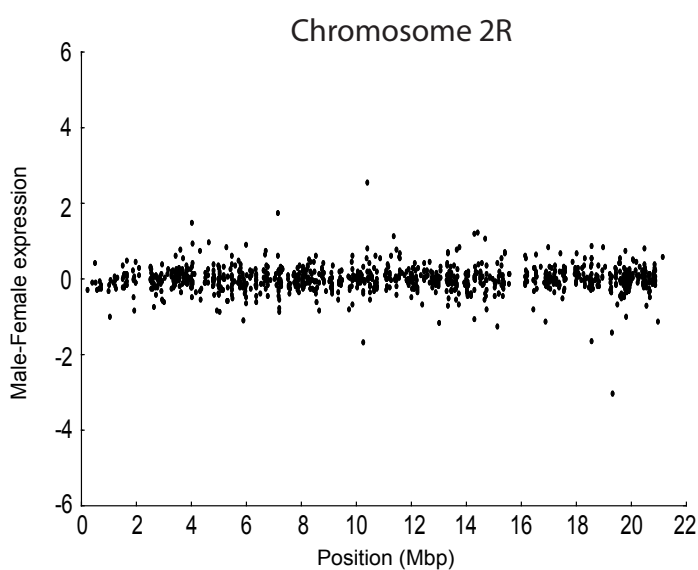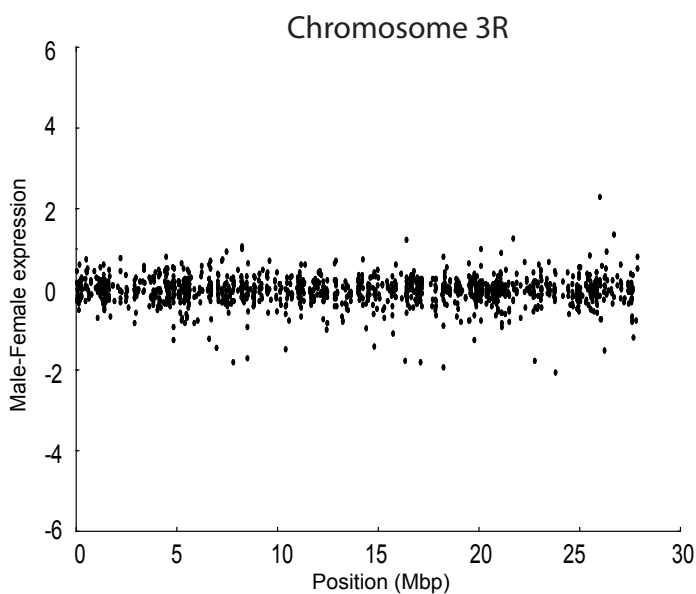

Supplement: Additional file 3: Figure S3 — Male minus female expression in salivary glands (log2) of all genes expressed in both sexes along chromosomes 2L, 2R and 3R. [file 1756-8935-6-35-S3.pdf]
